# Accessible areas in ecological niche comparisons of invasive species: Recognized but still overlooked

**DOI:** 10.1038/s41598-017-01313-2

**Published:** 2017-04-27

**Authors:** Huijie Qiao, Luis E. Escobar, A. Townsend Peterson

**Affiliations:** 1 Key Laboratory of Animal Ecology and Conservation Biology, Institute of Zoology, Chinese Academy of Sciences, Beijing, China; 2 Minnesota Aquatic Invasive Species Research Center, University of Minnesota, St. Paul, MN, 55108 USA; 3 Biodiversity Institute, University of Kansas, Lawrence Kansas, USA

## Abstract

Understanding biological invasions is crucial for their control and prevention. Specially, establishing whether invasive species operate within the constraint of conservative ecological niches, or if niche shifts occur at all commonly as part of the invasion process, is indispensable to identifying and anticipating potential areas of invasion. Ecological niche modeling (ENM) has been used to address such questions, but improvements and debate in study design, model evaluation, and methods are still needed to mature this field. We reanalyze data for Gray Squirrels (*Sciurus carolinensis*), native to North America, but invasive in Europe. Our main finding was that, when the analysis extent is established carefully based on analogous sets of environmental conditions, all evidence of niche shifts disappears, suggesting that previous reports of niche shifts for this species are artifacts of methods and interpretation, rather than biological reality. Niche conservatism should be tested only within appropriate, similar, environmental spaces that are accessible to both species or populations being compared, thus avoiding model extrapolation related to model transfers. Testing for environmental similarity between native and invaded areas is critical to identifying niche shifts during species invasion robustly, but also in applications of ENM to understanding temporal dimensions of niche dynamics.

## Introduction

Debate about the hypothesis that organisms tend to retain ancestral ecological niche traits or that those traits change only slowly (‘niche conservatism’) emerged 17 years ago^1^, but still has not seen consensus^2^. An emerging hypothesis that may solve this debate is that niche conservatism dominates over short-to-moderate time spans and at lower taxonomic ranks (genus and species), but less over longer time spans and at higher taxonomic ranks^3^. Many studies have sought to test this hypothesis quantitatively, such as via longitudinal tests examining distributional shifts from the Pleistocene to present (e.g., refs 4–6), cross-sectional tests comparing sister taxon pairs^7^, and comparisons of invasive species between native and invaded areas^8^. Ecological niche models (ENMs) have been the principal tool used to estimate niche dimensions and potential distributions of species, but have often been used uncarefully^9–11^. In recent evaluations, indices of niche similarity have been used in tandem with randomization tests and species-specific hypotheses of accessible areas to assess whether niches have been conserved or not^12^.

Using ENMs to generate potential distribution estimates for each population or species, calculating some level of niche similarity, and comparing to a null distribution of expectations are typical steps that recent studies have followed^13–16^. However, the premise for comparing two ENMs is that both are distributed across the same or similar regions; that is, in cases in which available environmental conditions differ absolutely between the species being compared, tests will either be inconclusive or will reach incorrect conclusions^3^. Several published niche comparisons for invasive species have not indicated the step of assessing whether available environmental conditions overlap between the species or populations being compared^8, 17–20^, making their reports of niche shift of uncertain credibility^3^.

As a result, several recent papers on species invasions have concluded that niches have shifted when available evidence suggests use of novel environments by invasive species in the invaded range^8, 17, 20, 21^, when those conditions could be unavailable or inaccessible in the native range^22–24^. This interpretation confounds the idea of fundamental niche (an evolved feature of the phenotype) with aspects of availability of conditions across real-world landscapes. On the other hand, ecological and evolutionary theory suggests that niche conservatism ought to be more common than niche shifts^25^. Hence, apparent niche shifts over short periods of time (e.g., during species invasions) should be analyzed carefully to assure that they are not consequences of analytical artifacts or incorrect assumptions or interpretations^3, 23, 26^. We note that this debate is not just a technical detail: if niches indeed shift easily during species’ invasions, predicting the geographic potential of invasive species will be much more difficult.

Conceptual explorations of observable ecological niches indicate a crucial point: inferences must consider the area accessible to the species (**M**) and the set of environments represented across that region, termed *η*(**M**) by Peterson *et al*.^27^, as the limits of the observable niche. That is, more formally, the existing fundamental niche **N**_F_* is the intersection of the true fundamental niche **N**_*F*_ with *η*(**M**), such that **N**_*F*_ * will always be a subset of **N**_*F*_, and any attempt to extrapolate **N**_*F*_ * into an estimate of **N**_*F*_ is perilous^28^. Based on this reasoning, changes in **M** will generate changes in the observable niche, even when the fundamental niche remains unchanged^27^. However, the question is whether the fundamental niches of two species or populations are equivalent (i.e., whether **N**_*F*1_ = **N**_*F*2_), if only **N**_*F*_ * **⊆** **N**_*F*_ is observable, any part of **N**_*F*_ not represented within **N**_*F*_ * is in some sense extrapolative, and generally unreliable for inference^28^. Hence, a revised question is whether the two niches are equivalent *given the set of environments available or accessible to each*, or whether **N**_*F*1_│*η*(**M**_1_) = **N**_*F*2_│*η*(**M**_2_). Any tests for niche similarity or difference lacking the conditional will run the risk of interpreting differences in environmental representation across accessible areas as niche difference^3^—we reiterate that a hypothetical change in the fundamental niche has important implications for the environmental and geographic potential of the species, whereas change in the existing niche is expected with any and every change in geographic (and environmental) setting.

**Figure 1:**
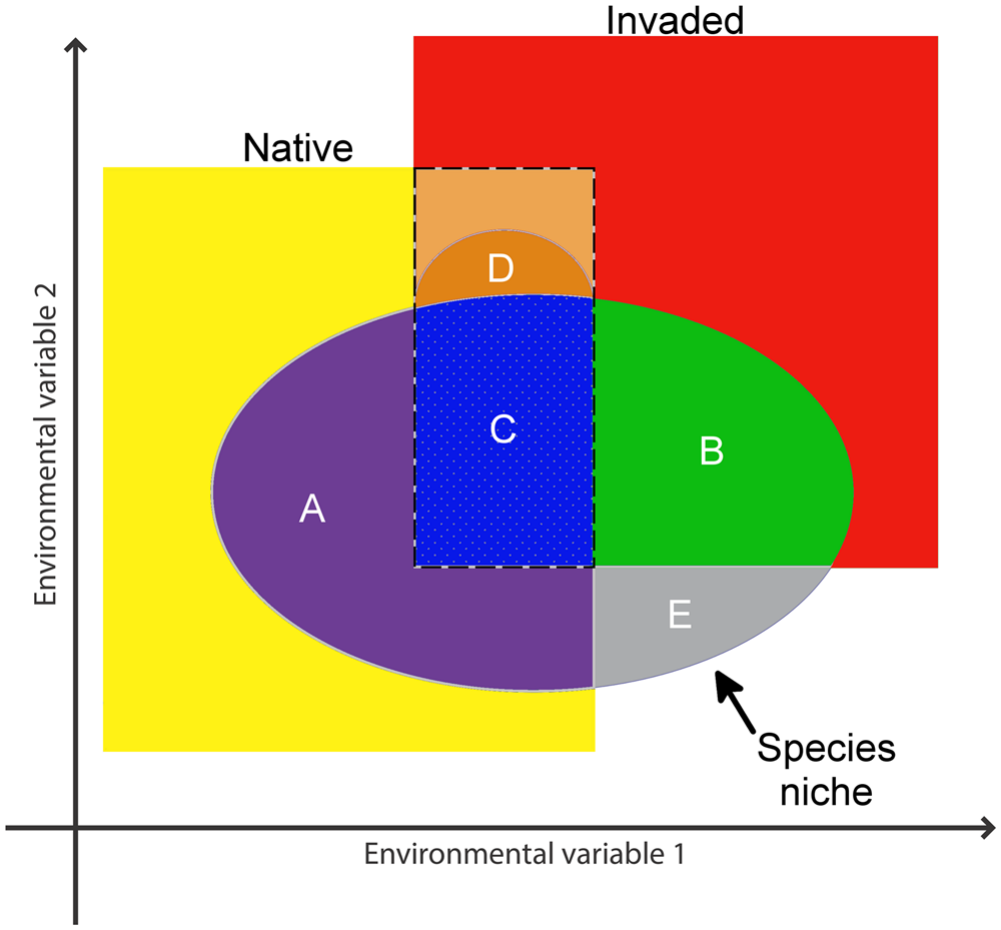
Framework of species’ ecological niches across accessible areas in native (yellow) and invaded (red) ranges. Portions of the niche are as follow: (**A**) native-range environments (purple): species using environments available only in the native range; (**B**) invaded environments (green): species using environments available only in the invaded range; (**C**) overlapping environments (dashed line): invaded-range populations using the same environments as in the native range (blue); (**D**) overlapping environments: species using novel environments in the invaded range (i.e., not used in the native range, even when available; dark orange); (**E**) Environments not available in either native or invaded ranges (=the unfilled niche). Similar environments available in both, the native and invaded ranges are indicated with a dashed line. Arrow indicates the species’ fundamental niche (**A**,**B**,**C**,**D**,**E**). Figure done using GIMP2. GIMP Team, GIMP 2.8.10, www.gimp.org, 1997–2016.

Peterson *et al*.^27^, and Owens *et al*.^28^, concluded that niche models are generally able to estimate robustly only **N**_*F*_ * or **N**_*R*_ (the realized niche), each only a portion of the fundamental niche (Fig. 1). Should we call the simple expansion of the realized niche across a broader and more environmentally diverse **M** area (i.e., during species invasion) a niche shift? We believe not, as the fundamental niche remains unchanged, and the distributional potential of the species similarly. Rather, in light of recent demonstrations of dramatic, biologically unrealistic extrapolation that occurs in transferring niche models from **M** to broader areas^28^, we propose that quantification and testing of niche shifts should be done only within the set of environments overlapping between the populations in native and invaded areas (Fig. 1). Genuine niche shifts occur when the two species or populations use different environments within these doubly accessible sets of conditions. When environments in the accessible areas of the two populations do not overlap, a null hypothesis of niche similarity cannot be rejected, and differences in realized niches must be considered as explainable as simple distributional expansion within the constraints of the same niche (Fig. 1).

To exemplify these ideas, we evaluate environments represented across native and invasive areas and associated accessible areas for one invasive species, to see how different they are. Our aim is to establish whether real niche shifts are demonstrably occurring, or we are possibly simply seeing what is best explained as new parts of the same fundamental niche. Differentiating between these two scenarios is crucial to understanding the invasion process and in turn to designing effective strategies for invasive species control. A genuine shift in the fundamental niche would reflect evolutionary adaptation of a species to novel environmental conditions, whereas occupying new portions of the same fundamental niche does not change the environmental or geographic potential of the species. We used a species and approaches from a recent article reporting niche shifts in Gray Squirrels (*Sciurus carolinensis*), a mammal native to North America that is invasive in the United Kingdom and elsewhere in Europe^20^. The original article tested whether Gray Squirrels introduced into Europe maintained the same niche as in its native range, and developed a global model using data from the native and invaded ranges to identify areas of potential expansion by this species. The authors reported niche shifts in the areas of introduction. Our reanalysis may provide a key consideration for further assessments of niche conservatism and niche shift of invasive species.

## Results

**Figure 2:**
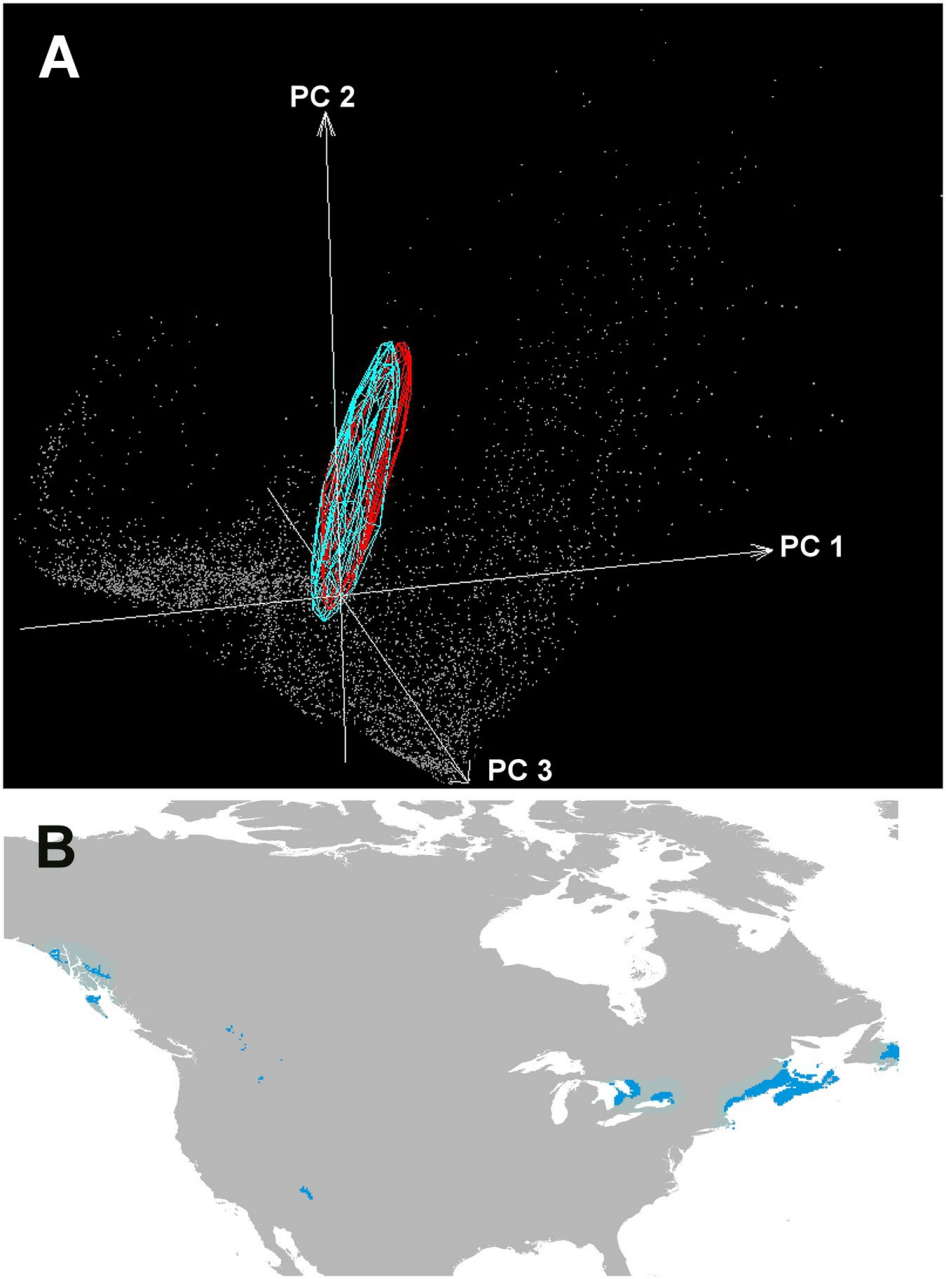
Analogous environments in native and invaded areas. Top: visualization of similar overlapping environments in native (blue convex polyhedron) and invaded (red convex-polyhedron) areas for Gray Squirrels. Note the many unshared environments across the native range (gray points). Axes are the first three principal components derived from the original climate layers. Bottom: geographic areas with analogous environments in the native range of North America (blue). Note the many unshared environments across the native range (gray areas). Top figure done using NicheA 3.0 [ref. 48]. Bottom figure done using ArcGIS 10.2 (ESRI, Redlands, CA, http://www.esri.com/).

We explored environmental characteristics of each study area, and found that the environmental conditions corresponding to invaded areas in the British Islands were completely contained within the native-range environments (Supplementary Material S1), at least with respect to the first three principal components. Environments shared with the British Islands covered only a small portion of conditions in North America (Fig. 2). In other words, based on three methods (i.e., MOP, NicheA, ExDet), we found no novel environments across the geographic area of the species’ invasion (Supplementary Material S2); this finding allowed us immediately to reject the scenario of novel portions of the species’ fundamental ecological niche becoming observable only on invaded areas (Fig. 1).

**Figure 3:**
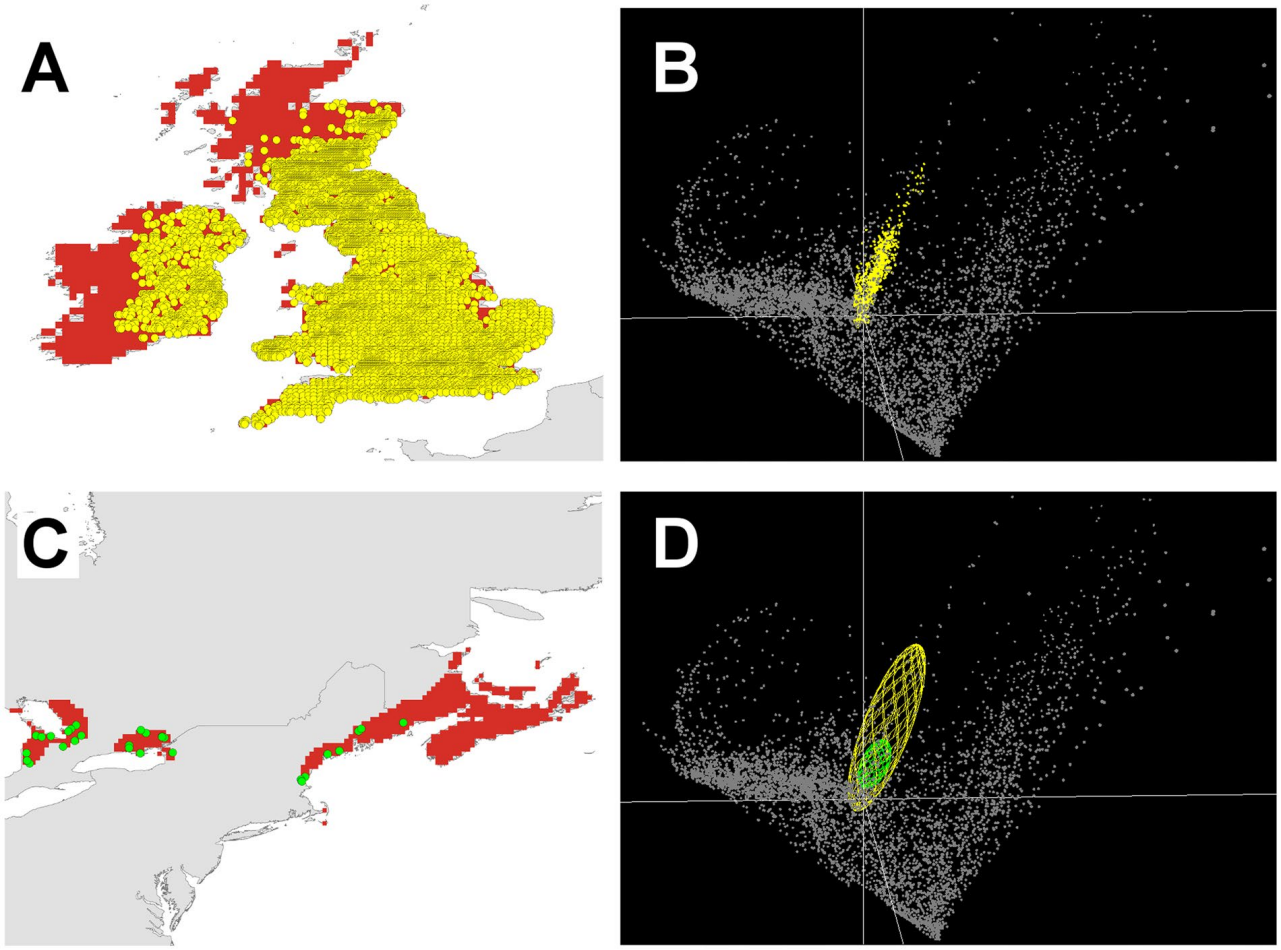
Gray Squirrel distribution. (**A**) Occurrences in geographic space (yellow points) in invaded areas (red). (**B**) Display in environmental space of corresponding occurrences from invaded areas (yellow points). (**C**) Geographic occurrences (green points) in the native range, restricted to areas with environments analogous between native and invaded range (red). (**D**) Display in environmental space of models from occurrences in the native (green ellipsoid) and invaded ranges (yellow ellipsoid) falling in environments analogous between native and invaded range. Notice the occurrences from native areas (green ellipsoid) nested within the ellipsoid corresponding to occurrences from the invaded areas (yellow ellipsoid) in the environmental space (**D**). Background values are show as gray. Left figures done using ArcGIS 10.2 (ESRI, Redlands, CA, http://www.esri.com/) and right figures done using NicheA 3.0 [ref. 48].

**Figure 4:**
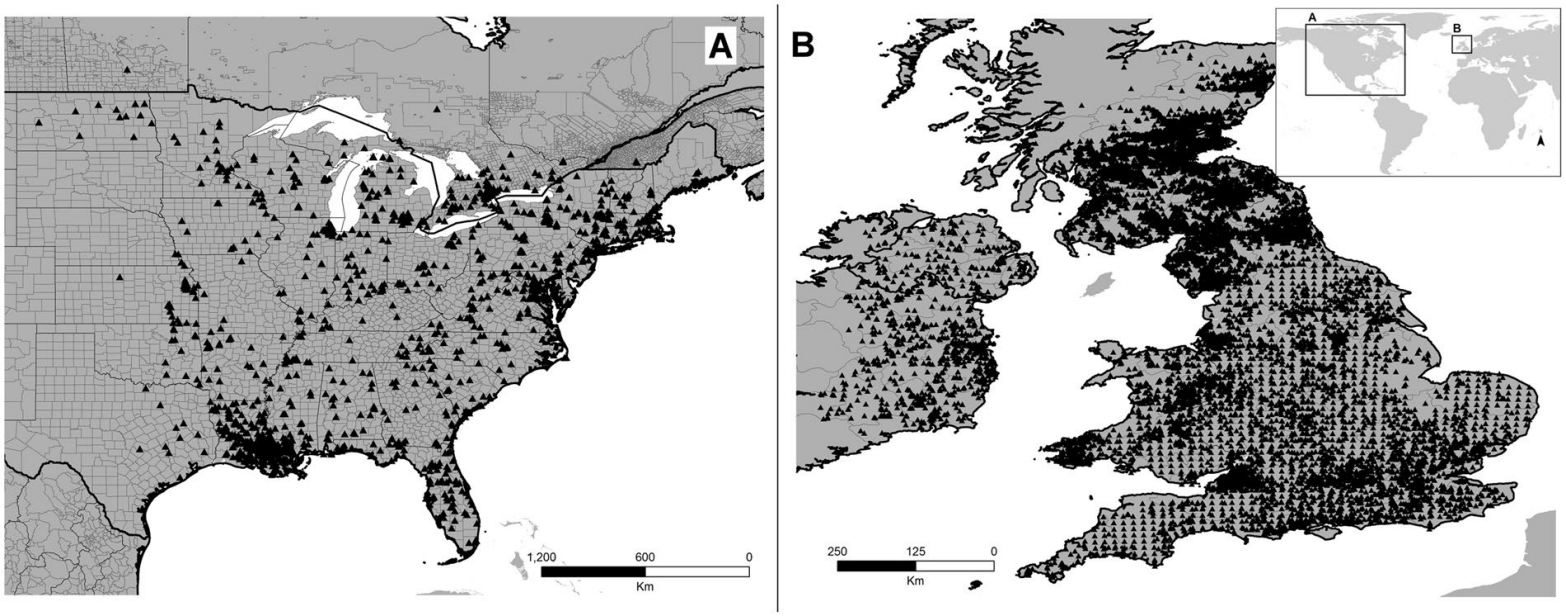
Study area and Gray Squirrel occurrences (black triangles) used to compare the native (**A**) North America) and invaded (**B**) United Kingdom & Ireland) ranges. Insert shows the geographic location of the study areas. Figure done using ArcGIS 10.2 (ESRI, Redlands, CA, http://www.esri.com.

Although the original study^20^ documented differences in climate signature between native and introduced populations, we found no evidence of niche shifts when models from occurrences falling in analogous environments were compared (Fig. 2). Indeed, we found higher niche similarity of the Jaccard index in analogous environments when compared with the models including all the occurrences and environments in North America (Table 1). When considering occurrences from analogous environments (i.e., geographic space; maps in Fig. 3), niche models from the native and invaded populations had similar shape and position, but not size (i.e., environmental space; ellipsoids in Fig. 4); this difference derived from the relatively few occurrences available from the restricted areas presenting overlapping environments in North America (*n* = 31 occurrences), compared with many more from invaded areas (*n* = 5956 occurrences).

**Table 1:** Examination of the environmental similarities between native and invasive Gray Squirrel (*Sciurus carolinensis*) populations.

Areas	Jaccard index	Warren *D* index	Warren *D* index (*α* = 0.05)
Original areas and points	0.50	0.38	Similar^a^ Similar^b^
Only points in areas with analogous environments	0.01	0.13	Dissimilar^a^ Similar^b^

^a^British Islands occurrences versus North America background.

^b^North America occurrences versus British Islands background.

Background similarity tests across the entire (original) study areas (i.e., the whole native and invaded ranges) showed niche similarity between populations of North America and the British Islands (*P* < 0.001). However, when analyses were restricted to occurrences and areas falling in analogous environments the metric was inconclusive (Fig. 3): a null hypothesis of niche similarity could not be rejected in one of two evaluations (Table 1). Analyses of occurrences distribution in the environmental space, using the ecospat tool of Broennimann *et al*.^29^ showed considerable overlap in the environmental conditions occupied in the native and invaded ranges by Gray Squirrel. This evaluation showed sampling bias of occurrence records in the form of high densities of records in environmental space. The niche overlap, however, fell within the 95% confidence limits of the null distributions, leading to non-rejection of the hypothesis of niche similarity (Supplementary Material S3). Hence, in light of these tests (Jaccard similarity, background similarity, and similarity in environmental space), even the evidence for niche similarity was contingent on available occurrences and comparisons from uncontrolled environments, and extrapolation into non-analog sets of conditions.

## Discussion

Ecological niche modeling has seen broad adoption in ecology, biogeography, invasion biology, and pest management, and has been applied to questions of biological dynamics of different organisms and ecosystems across diverse spatial and temporal scales worldwide^27, 30, 31^. Species’ geographic distributions are structured by combinations of biotic factors, abiotic factors, and the dispersal potential of the species^32^. Ecological niche modeling focuses on abiotic dimensions^33^, but its efficacy is affected by dispersal^34^; hence, ideally, model outputs should be assessed and interpreted in environmental spaces, but keeping in mind the geographic context of the analysis^27, 35^. Currently, methods for niche model comparison suffer from considerable lack of standardization^23^, or are cast in geographic dimensions^14^, which has inserted considerable variation into results of studies. Although one approach does indeed conduct comparisons in environmental space^29^, it does not automatically control for whether or not models have had to be transferred to make them comparable. Guisan *et al*.^23^ reviewed ecological niche modeling studies for 180 invasive species, finding that niche shifts reported in most cases depended on questionable study design; they concluded that establishing environmental analogy should be the *sine qua non* in studies of niche shift in biological invasions.

Di Febbraro *et al*.^20^ pointed out an important issue as regards to the catastrophic effects of Gray Squirrels on native squirrel populations in the United Kingdom, and the current risk of spread to other areas of continental Europe, all via the pet trade, but also concluded that niche shifts have played a role in facilitating the invasion process. We came to opposite conclusions as regards to niche shifts during this invasion: analysis of environmental data across the entire native area generates “noise” in comparisons with the relatively narrow set of environments manifested in the invaded areas^34^. As a result, Di Febbraro *et al*.^20^ concluded that niche shifts had occurred between native *versus* invasive populations, but their result appears to be an artifact of the different environments manifested in the two areas more generally. In fact, no genuinely novel environments were involved at all in the invasion: the misinterpretation was perhaps caused by comparison of a very rich environmental universe from a broad geographic extent (the native area) against a much more restricted climatic range in the invaded areas (see Fig. 4 in Di Febbraro *et al*.^20^).

We found that, when models from both populations were compared across broad and diverse environmental backgrounds (i.e., the two distributional areas), the *D* index was consistent; however, when evaluations were restricted to analogous environments only, the same metric yielded mixed signals. This result illustrates how sensitive these metrics are to differences in environmental representativeness of available occurrences and distributional areas. Di Febbraro *et al*.^20^ supported the niche shift conclusion based on identity tests and visualization of different positions of the clustered occurrences in the environmental space (see Fig. 4 in their manuscript). However, the first approach is highly subject to Type I error^3^, and the second lacks statistical support and is influenced by sampling bias and **M** hypotheses (see Supplementary Material S1). Our study aimed to replicate the experiments of Di Febbraro *et al*.^20^; however, when assessing the potential of species to invade a region, the entire species’ range including native and invasive populations should be considered in model calibration^24^. For example, Gray Squirrels are reported to be invasive in regions of Canada, Ireland, Italy, Pitcairn, South Africa, and the United Kingdom^36^, so more informed forecasts could be obtained considering all the established populations. Additionally, we caution on the use of Broennimann *et al*.^29^ method to assess niche similarity in environmental space as it may be impacted by the sampling bias and the background available for comparisons.

Several recent contributions showed important impacts of selection of **M** extent on the model output, thus showing that results of ecological niche models are scale dependent^34, 37^. Some methods for a delimitation of model calibration areas **M** include consideration of biomes where the species occur^23^, dispersal estimates from the invasion process^38–40^, dispersal potential in the native range based on occurrences^41^, and areas defined arbitrarily for exploratory analyses of poorly known-species^42^. Thus, selection of the **M** hypothesis should be supported by the data or biogeographic literature on the species, instead of using administrative boundaries. However, such approaches are limited to geographic dimensions. Here we propose that areas with analogous overlapping environmental conditions be the focus of attention in assessment of niche shifts.

Because existing approaches do not take precautions against extrapolative situations^14^, here, we explicitly limited comparisons of niche to shared sets of environments. Di Febbraro *et al*.^20^ explored niche shifts via models calibrated across the invaded areas; we showed that models calibrated in invaded areas would likely yield weak inferences when transferred across the entire native area, owing to massive opportunity for extrapolation^28, 43^. Our findings of a complete nesting of the climate from the invaded range within climates in the native range (Supplementary material S1) were also noted in the original study but were neglected (Fig. 4 in Di Febbraro *et al*.^20^). Model transfers from invaded to native areas replicated here reflect effects of how particular algorithms extrapolate onto novel environmental conditions (see black areas in Supplementary Material S2).

This tendency to conclude niche shift based on incomplete or biased evidence has appeared frequently (e.g., refs 8, 17 and 19), as pointed out previously by Peterson^3^. It should be considered carefully when ecological niche modeling is used to evaluate risk of pest invasion, particularly among areas differing markedly in extent or diversity of environments^28^. Hence, in this contribution, we have explored means of testing niche similarity or difference without need for model transfers, which too-frequently extend to extrapolative situations. While the background similarity test is a two-way test of niche similarity^14^, test of niche similarity for invasive species should be more relevant in one direction: to assess if the invasive population has a niche more similar to the native niche than expected by chance. We note that our approach is presently implemented in three dimensions only, whereas niche differentiation can be manifested in any of the dimensions that make up the full dimensionality of the environmental space; we also note that our use of principal components analysis to simplify environmental space could be replaced by any number of alternative approaches that may be more appropriate under certain circumstances^44, 45^.

We argue that conclusions of niche conservatism *versus* niche shift should be drawn based on comparisons only within environments accessible to both species. If environmental spaces in the two accessible areas are non-overlapping, tests of niche similarity will be (and should be) inconclusive, because the two species are observable only against distinct environmental backgrounds. Without this precaution, tests are, in effect, interpretations of patterns of model extrapolation, which is known to be unpredictable and not realistic in terms of physiological responses of organisms^28^. Restricting areas of analysis as we recommend certainly involves lower statistical power owing to reduced sample sizes, but should be far less prone to spurious conclusions of niche shift. Indeed, evaluation of non-analogous environmental conditions should be crucial not only in invasion ecology but also in studies of climate change impacts on the distribution of species; ecological niche modeling applications to assessing distributional responses of species to warming climates, lacking evaluation of novel climates, should be considered with caution. This balance between appropriateness of conclusion and statistical power constitutes an important next step in development of mature methodologies for these tests.

## Methods

We chose a study area extent, occurrence data, and environmental variables, following the previous publication^20^, but excluding the Piedmont area in Italy, as those occurrences were not made available (Fig. 4). Occurrences were resampled to one occurrence point per grid cell at 2.5′ resolution. The area across which models are calibrated affects performance of ecological niche models based on correlative algorithms requiring background data or pseudo-absence data for calibration^34^. Thus, for a replicate experiment our **M** was based on previous evaluations for the Americas and Europe^20^: we included the area from Central America (Nicaragua, Honduras, El Salvador) and the Greater Antilles (Jamaica, Cuba, Dominican Republic, Haiti) north to northern Canada and Alaska; in Europe, we focused on Ireland and the United Kingdom.

Occurrence points for both native and invasive populations were obtained from the Global Biodiversity Information Facility (http://www.gbif.org), Mammal Networked Information System (http://manisnet.org/), and the online specimen databases of the American Museum of Natural History (http://www.amnh.org/) and U.S. National Museum of Natural History (http://www.mnh.si.edu/). We removed duplicates and included 1754 occurrences falling within the native geographic range^46^ and 16,636 in the United Kingdom and Ireland.

Considering that our aim was to compare two modeling approaches, as environmental information we used the same data layers as in the original article^20^: WorldClim climate data at 2.5′ resolution^47^, including mean temperature of the wettest quarter, mean temperature of the coldest quarter, precipitation of the wettest quarter, and precipitation of the coldest quarter. These data were checked for nonlinear relationships and converted to principal components^27^, and environments on each continent were extracted to establish overlap with respect to the first three components (Supplementary material S1), which jointly explained 97% of the variance in the data. Components 1, 2, and 3 were displayed and analyzed using NicheA version 3.0, an open-access software platform that allows design, exploration, and analysis of geographic and environmental spaces simultaneously^48^. We also identified areas with non-analogous environmental conditions using three methods. First, we used NicheA to identify environmental combinations shared between the two continents. NicheA generates two convex-polyhedrons each around the range of climatic conditions in the native and invaded range respectively, and then determines the similar environments in the form of conditions overlapping between both polyhedrons; overlapping environments were projected from environmental space to geographic space to determine the study area and subsets of occurrences to be used to establish presence or absence of niche shifts. Secondly, we used the Mobility-Oriented Parity (MOP) metric that measures environmental similarity between native and invaded areas^28^. MOP estimates Euclidean distances between the native and invaded ranges in multivariate environmental spaces; MOP then identifies and excludes the values outside of the environmental range of the calibration region. The similarity values of MOP are comparable to the distances estimated by the multivariate environmental similarity surface (MESS), but the latter measures distances to the centroid, rather than the near edge, of the reference environmental background^49^. Finally, we employed ExDet to identify similar or novel environments between native and invaded area^50^. ExDet measures Mahalanobis distances between environmental conditions in the native and invaded areas and identifies areas presenting conditions outside the univariate range of values in the other area (i.e., novelty Type 1). ExDet also identifies the most influential environmental variables leading to non-analogous environments between the areas compared^50^.

We used NicheA to assess niche similarity via the Jaccard index^51^ in analogous and non-analogous multidimensional environments based on components 1, 2, and 3 [ref. 48]. We also used ENMTools 1.4.3 [ref. 12] to develop background similarity tests of niche similarities in geographic dimensions. We measured the *D* similarity index to compare similarity between Maxent models generated using occurrences available from the native range (North America) against occurrences in the invaded range (i.e., British Islands), restricted to occurrences falling in analogous environments between European and American accessible areas. This analysis was also done across the entire (original) areas as in Di Febbraro *et al*.^20^. We compared observed similarity between the invasive population and the native range against a null distribution generated from comparisons of the invasive population and occurrence points placed at random within the native population in analogous environments^12^. Specific features in ENMTools were use of binary outputs from minimum-training presence, 100 replicates to build the null distribution, *α* = 5%, and sample sizes for background points matching available numbers of points, using the principal components (see above) to summarize environments. Additionally, we used ecospat^29^ to evaluate niche similarity in environmental dimensions, using the first two principal components from the original environmental variables, occurrences from native and invaded ranges with analogous environments, and identifying the 50% and 100% of environmental conditions available in each range.

## Electronic supplementary material

Supplementary Material
