# Supplementary material for: Accessible areas in ecological niche comparisons of invasive species: Recognized but still overlooked
Source: Sci Rep. 2017 Apr 27;7:1213. doi: 10.1038/s41598-017-01313-2 (PMC5430674; doi:10.1038/s41598-017-01313-2)
Supplement: Supplementary file 1 — Supplementary Material [file 41598_2017_1313_MOESM1_ESM.pdf]

Title

Accessible areas in ecological niche comparisons of invasive species: Recognized but still overlooked

Authors

Huijie Qiao[1†], Luis E. Escobar[2†*], A. Townsend Peterson[3]

Affiliation

[1]Key Laboratory of Animal Ecology and Conservation Biology, Institute of Zoology, Chinese Academy of Sciences, Beijing, China

[2]Minnesota Aquatic Invasive Species Research Center, University of Minnesota, St. Paul, MN, USA 55108

[3]Biodiversity Institute, University of Kansas, Lawrence Kansas, USA

*Corresponding author: Luis E. Escobar, Department of Fisheries, Wildlife and Conservation Biology, University of Minnesota, 135 Skok Hall, 2003 Upper Buford Circle, St. Paul, MN 55108-6074, (+1) 612-301-1821. E-mail: lescobar@umn.edu

Supplementary Material S1. Environmental space showing climatic conditions available in the native (blue) and invaded range (red). Axis are principal components one (PC1), two (PC2), and three (PC3) from the original climatic variables (see methods).

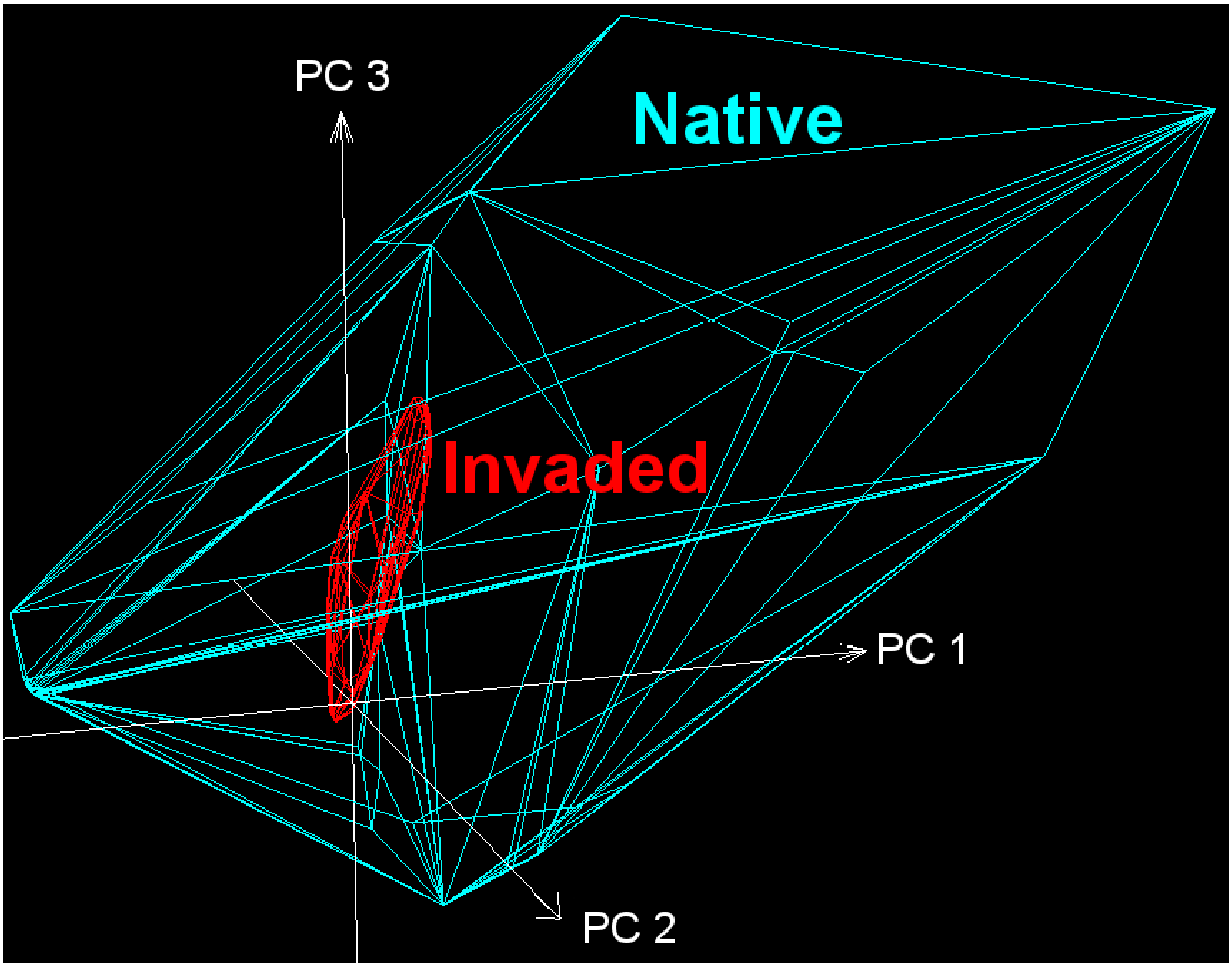

Supplementary Material S2. Assessment of environmental similarity between native and invaded ranges.

S2.1 Assessment of environmental similarity using the NicheA and MOP.

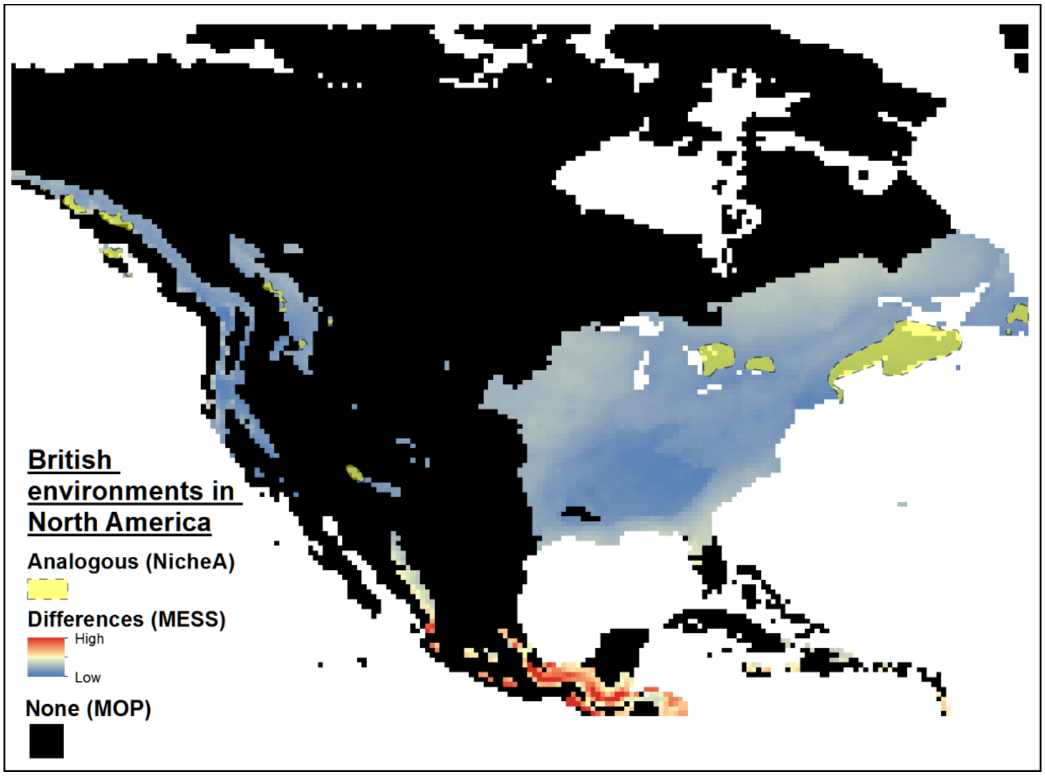

Map of the mobility-oriented parity (MOP) metric to evaluate environmental similarity between the Gray Squirrel (*Sciurus carolinensis*) native range in North America and its invaded range in British Islands. Only limited areas of North America contained environments represented in the British Islands (yellow), while a broad portion of North America has environments different from those on the British Islands (black). Figure done using ArcGIS 10.2 (ESRI, Redlands, CA, http://www.esri.com/).

S2.2 Assessment of environmental similarity using the ExDet.

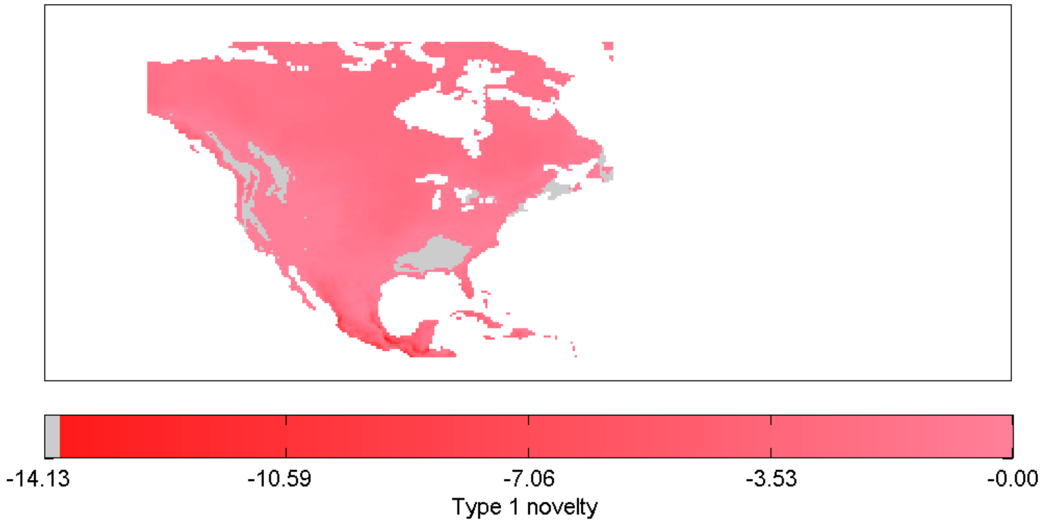

The non-analogous environments (conditions outside the range of individual covariates) present in North America but absent in the British Islands are denoted in red. The range of values denote the degree of type 1 novelty (Mesgaran et al., 2014). Areas with environments similar between native and invaded ranges are denoted in gray. Figure done using ExDet (Mesgaran et al., 2014).

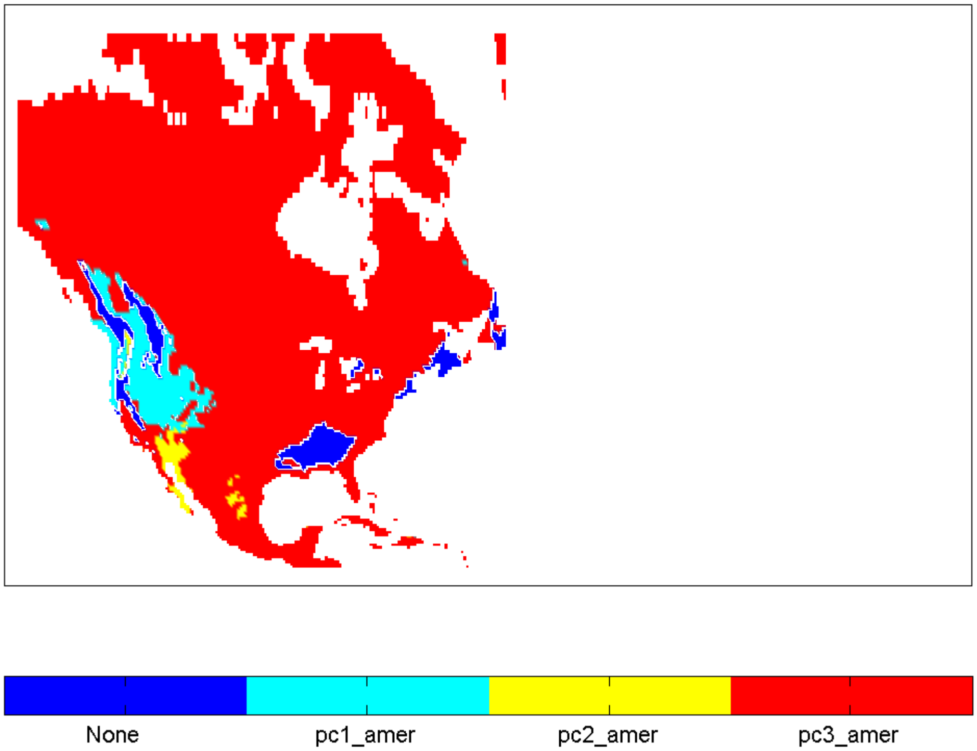

Type 1 novelty from the native range measured by variable. The analogous environments, conditions present in North America and also present in the British Islands, were identified according to the environmental variable responsible for such novelty using the most influential covariates leading to Type 1 novelty analysis (MIC for NT1) (Mesgaran et al., 2014). Conditions in North America outside the range of values in the British Islands were identified for principal component 1 (light blue), two (yellow), and three (red). Environmental conditions shared between native (North America) and invaded range (the British Islands) are denoted in dark blue. Figure done using ExDet (Mesgaran et al., 2014).

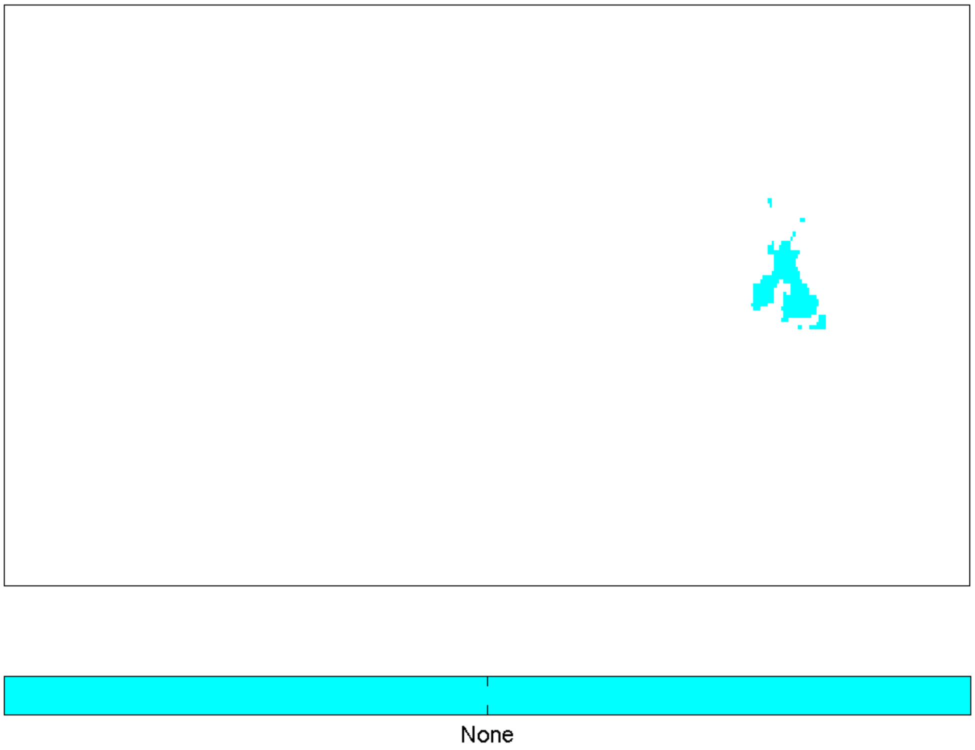

Pixel cell representing Type 1 novelty from the British Islands measured by ExDet. Analogous environments (conditions inside the range of individual covariates) present in the British Islands and also in North America are denoted in light blue. Novelty environments should be denoted in a range of red color (Mesgaran et al., 2014). Notice that all environments are analogous, and that no non-analogous conditions were found in the British Islands. In other words, all the environmental condition in invaded range in the British Islands can be found in native range in North America. Figure done using ExDet (Mesgaran et al., 2014).

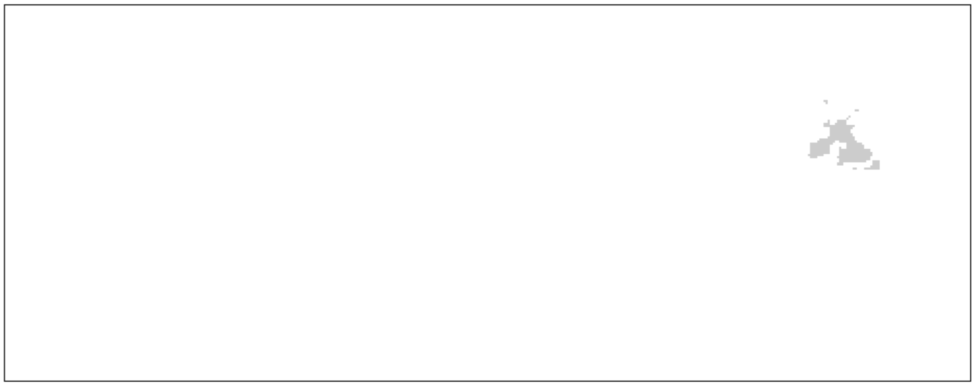

Pixel cells representing Type 1 novelty from the British Islands measured by variable. All environmental conditions in Europe were found in the native range of North America (gray) as identified by the most influential covariates leading to Type 1 novelty analysis (MIC for NT1) (Mesgaran et al., 2014). Figure done using ExDet (Mesgaran et al., 2014).

Supplementary Material S3. Niche similarity comparisons of the gray squirrel (*Sciurus carolinensis*) in environmental space using the ecospat tool.

.

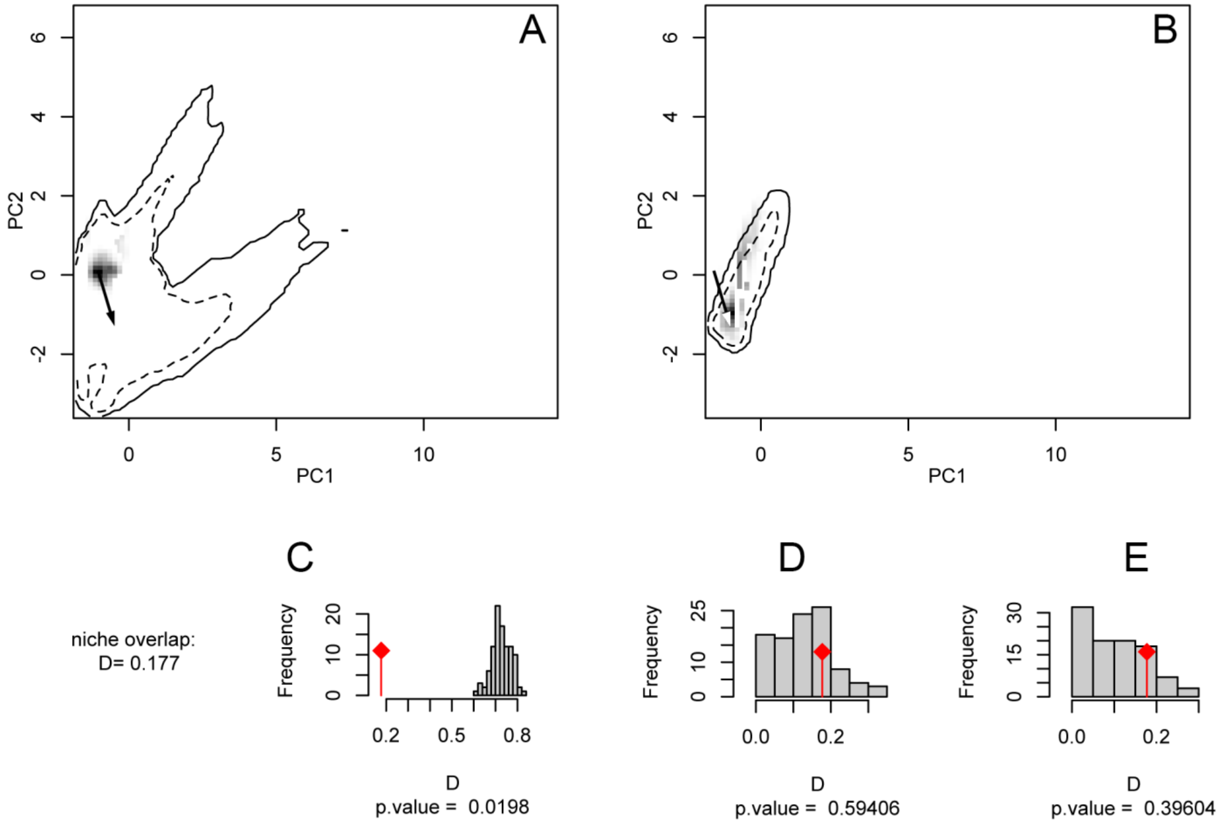

Panels A and B represent the gray squirrel occurrences along the first two axes of the PCA in the native (i.e., North American) and invaded range (i.e., British Islands), respectively. Gray shading shows the density of the occurrences of the species by cell. The solid and dashed contour lines illustrate, respectively, 100% and 50% of the available (background) environment. The arrows represent the distribution of the centroid of occurrences from North America and British Islands. Histograms C to E show the observed niche overlap *D* between the two ranges (bars with a diamond) and simulated niche overlaps (gray bars) on which tests of niche identity (C), niche

similarity of North American to British Islands (D), and niche similarity of British Islands to North America (E) are calculated from 100 replicates.
